# Prevalence, Predictors, and Early Outcomes of Post-operative Delirium in Patients With Type A Aortic Dissection During Intensive Care Unit Stay

**DOI:** 10.3389/fmed.2020.572581

**Published:** 2020-09-25

**Authors:** Shining Cai, Xiaomin Zhang, Wenyan Pan, Jos M. Latour, Jili Zheng, Jun Zhong, Jian Gao, Minzhi Lv, Zhe Luo, Chunsheng Wang, Yuxia Zhang

**Affiliations:** ^1^Department of Nursing, Zhongshan Hospital, Fudan University, Shanghai, China; ^2^Nursing School, Fudan University, Shanghai, China; ^3^Faculty of Health: Medicine, Dentistry and Human Sciences, School of Nursing and Midwifery, University of Plymouth, Plymouth, United Kingdom; ^4^Department of Biostatistics, Zhongshan Hospital, Fudan University, Shanghai, China; ^5^Department of Cardiac Surgical Intensive Care Unit, Zhongshan Hospital, Fudan University, Shanghai, China; ^6^Department of Cardiac Surgery, Zhongshan Hospital, Fudan University, Shanghai, China

**Keywords:** type A aortic dissection, delirium, incidence, risk factors, early outcomes

## Abstract

**Objectives:** The aim of this study was to investigate the prevalence and explore the predictors and early outcomes of post-operative delirium (POD) in patients with type A aortic dissection (AAD) during intensive care unit (ICU) stays.

**Methods:** We retrospectively reviewed the records of 301 patients with AAD who underwent surgical treatment in our institution from January 2017 to December 2018.

**Results:** Delirium developed in 73 patients (24.25%) during the ICU stay. Patients with lower estimated glomerular filtration rates [odds ratio (OR) 0.84, 95% CI 0.74–0.94, *p* = 0.003], post-operative midazolam use (OR 2.37, 95% CI 1.33–4.23, *p* = 0.004), and post-operative morphine use (OR 1.87, 95% CI 1.07–3.29, *p* = 0.029) were more susceptible to developing POD. Patients who developed POD had a longer ICU stay (11.52 vs. 7.22 days, *p* < 0.001) and hospital stay (23.99 vs. 18.91, *p* = 0.007) with higher hospitalization costs (48.82 vs. 37.66 thousand dollars, *p* < 0.001) than those without POD. The in-hospital mortality rate was higher in the delirium group, but the difference was not significant (6.85 vs. 4.82%, *p* = 0.502).

**Conclusions:** The incidence of POD in patients with AAD was high and was associated with renal dysfunction and the use of midazolam and morphine. POD was associated with poor early outcomes, suggesting the importance of early screening, such as for renal dysfunction, and prevention by using sedation scales to minimize the use of midazolam and morphine in these patients.

## Introduction

Post-operative delirium (POD) is common in patients undergoing elective cardiac surgery, including aortic valve replacement and coronary artery bypass grafting (CABG). The reported incidence of elective cardiac surgery varies between 3 and 17.3% (1–4). Type A aortic dissection (AAD) is a life-threatening condition requiring surgical intervention (5, 6). As both the disease itself and intraoperative deep hypothermic circulatory arrest might cause ischemia to the cerebral circulation and nervous system, patients with AAD might have a greater risk of developing neuropsychiatric complications than patients undergoing other cardiac surgeries (7, 8). Due to the stress of surgery and the typical environment, post-operative patients in intensive care units (ICUs) have higher incidence rates of POD than other patients (9). Although diagnostics and surgical techniques have significantly improved, the post-operative neurological morbidity rate in AAD patients remains high (10). POD is associated with several negative outcomes, including elevated morbidity and mortality, prolonged ICU stay, and extra medical expenses (11–13).

POD is a common neuropsychiatric disorder in AAD patients, but studies focusing on the potential effects of POD among these patients are sparse. Several studies have demonstrated a high prevalence of POD among these patients and raised concerns about delirium prevention in this population. However, the risk factors reported was diverse, and these studies are limited by small sample size (10, 14, 15). Due to the particularity of disease and surgery, the characteristics of POD in AAD patients might be quite different from other patients of cardiac surgery. The predictors and prognosis still need to be further explored to offer evidence for POD prevention, and help find out the perniciousness of POD in these patients. In this study, we aimed to explore the prevalence, predictors, and early outcomes of POD in patients with AAD during the ICU stay.

## Materials and Methods

### Design

This is a retrospective cohort study. The study has been reported following the STROBE guidelines for observational studies (16). Ethical approval was obtained from the institutional review board. The requirement for informed consent was waived because of the retrospective nature of the study.

### Setting

This study was conducted in a 39-bed cardiac surgery ICU of a tertiary hospital. The cardiac surgery ICU admits ~4,800 post-operative patients per year, including 150–200 patients with AAD.

### Study Population

**Figure 1:**
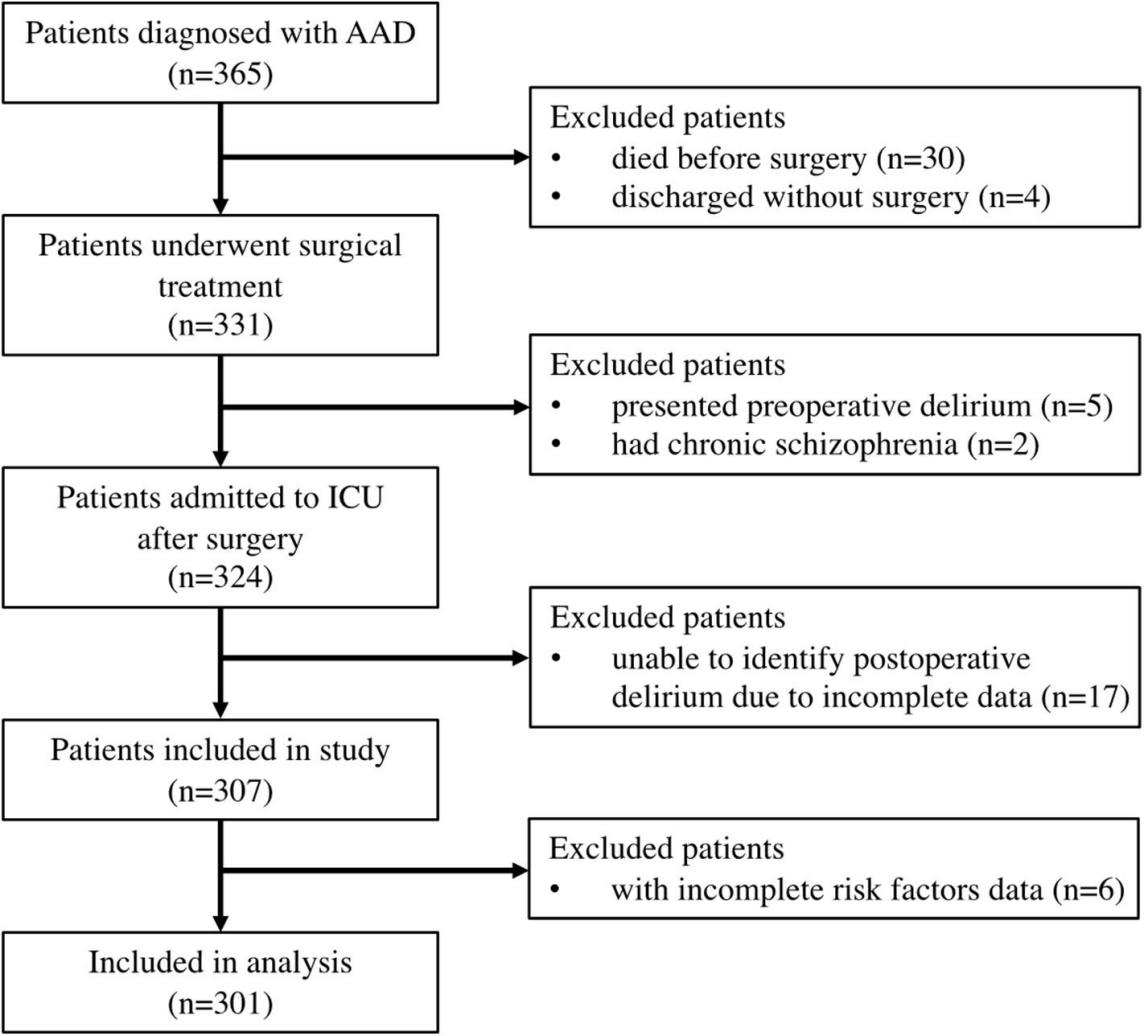
Flow diagram of patient selection.

We retrospectively reviewed patients who underwent surgical treatment for AAD from January 2017 to December 2018. The diagnosis of AAD was determined according to CT angiography. We excluded patients who had delirium or chronic schizophrenia before surgery. We also excluded patients who were unable to be diagnosed with POD due to missing data. Finally, 301 patients were included in this study (Figure 1).

### Data Collection

The primary outcome was POD, which was defined as a minimum of one positive assessment using the Confusion Assessment Method–ICU (CAM-ICU) tool during the ICU stay. The CAM-ICU defines delirium if at least two features of the CAM-ICU are present (Features 1 and 2 OR 3 and 4) (17, 18). In addition, we also used treatment with olanzapine as a proxy for the diagnosis of delirium because in the ICU of our hospital, we used olanzapine only to treat delirium.

The pre-operative data were retrospectively reviewed and analyzed. Surgery within 24 h from the onset of symptoms was considered emergent surgery. In-hospital mortality was defined as death occurring during the post-operative hospital stay. The estimated glomerular filtration rate (eGFR) was used to assess renal function after surgery. The eGFR was classified into four groups: normal (eGFR >90 ml/min/1.73 m^2^), mild (eGFR 60–90 ml/min/1.73 m^2^), moderate (eGFR 30–60 ml/min/1.73 m^2^), and severe (eGFR <30 ml/min/1.73 m^2^).

### Operative Technique

All operations were performed through a mid-sternotomy. Total arch replacement combined with stented elephant trunk (SET) implantation was the primary surgical strategy in our hospital. Other techniques used along or in combination were the Bentall procedure, David I procedure, or CABG. All patients received standard transcutaneous cerebral oximetry monitoring and transesophageal echocardiography during surgery.

### Statistical Analysis

The primary end point is the incidence of POD. The rule of thumb in logistic modeling is that a minimum of ten events per predictor variable should be achieved based on simulation studies (19). We used the lowest incidence 34% (14) and ten valuables in logistic model to estimate the sample size of 294.

Data are presented as the mean and SD or median (interquartile range) for continuous variables and as percentages for categorical variables. We log transformed the N-terminal prohormone of brain natriuretic peptide (NT-proBNP) level because it had a right-skewed distribution. Normally distributed continuous variables were compared using one-way ANOVA. The Pearson χ^2^-test was applied to all categorical variables. Logistic regression models were used to investigate univariable and multivariable risk factors for POD. In the logistic regression analysis, potential predictors of POD were tested in a univariable fashion, and variables with *p-*values < 0.1 were included in the multivariable analysis. In the multivariable analysis, a backwards stepwise model was used. The results of the logistic regression analysis are presented as odds ratios (ORs) with the corresponding 95% CIs. The analyses were performed using SPSS version 22.0 (IBM, New York, NY) and R statistical software (R, version 3.5.1; R Project).

## Results

### Patient Characteristics

A total of 365 cases have been reviewed and after exclusion 34 patients who did not receive surgery, seven patients who presented with pre-operative delirium or had chronic schizophrenia, 17 patients with incomplete data to identify POD, and six patients with incomplete risk factor data. Data from 301 cases were analyzed (Figure 1).

The patients were divided into two groups according to whether they developed POD during the ICU stay. The demographic characteristics and perioperative factors of the non-delirium group and delirium group are shown in Table 1. The mean participant age was 50.66 (SD 12.24), and 79.40% were males. The incidence of POD was 24.25% in awake AAD patients after surgery. The incidence of POD was 23.83% (56/235) in males, whereas in females, the incidence was 25.76% (17/66). Patients with delirium had more post-operative blood transfusions, higher eGFRs, and higher blood urea nitrogen, NT-proBNP, and blood calcium levels than those without delirium. In addition, more patients in the delirium group received midazolam, dexmedetomidine, and morphine post-operatively than those in the non-delirium group (Table 1).

**Table 1:** Demographic characteristics and perioperative factors of delirium and non-delirium group.

**Variables**	**Total (*n* = 301)**	**Non-delirium group (*n* = 228)**	**Delirium group (*n* = 73)**	***P*-value**
**Demographic characteristics**				
Age, years	50.66 ± 12.24	50.46 ± 12.32	51.27 ± 12.08	0.627
Male, *n* (%)	235 (78.01)	179 (78.51)	56 (76.71)	0.747
Hypertension, *n* (%)	202 (67.11)	151 (66.23)	51 (69.86)	0.668
Diabetes mellitus, *n* (%)	12 (3.99)	11 (4.82)	1 (1.37)	0.305
Nicotine use, *n* (%)	53 (17.61)	36 (15.79)	17 (23.29)	0.159
Alcohol use, *n* (%)	16 (5.32)	10 (4.39)	6 (8.22)	0.231
**Surgical details**				
Emergency operation, *n* (%)	70 (23.26)	53 (23.25)	17 (23.29)	0.994
Total arch replacement, *n* (%)	280 (93.02)	212 (92.98)	68 (93.15)	0.961
Stented elephant trunk, *n* (%)	269 (89.37)	203 (89.04)	66 (90.41)	0.740
Bentall procedure, *n* (%)	37 (12.29)	24 (10.53)	13 (17.81)	0.099
David I procedure, *n* (%)	24 (7.97)	15 (6.58)	9 (12.33)	0.114
CABG, *n* (%)	13 (4.32)	8 (3.51)	5 (6.85)	0.222
Duration of surgery, min	476.91 ± 89.65	471.92 ± 84.96	492.49 ± 102.02	0.088
CPB time, min	198.34 ± 42.69	196.72 ± 40.42	203.40 ± 49.07	0.245
Aorta cross-clamp time, min	114.86 ± 30.16	114.61 ± 29.69	115.64 ± 31.79	0.798
Intraoperative blood transfusion, *n* (%)	263 (87.38)	197 (86.40)	66 (90.41)	0.425
**Post-operative factors**				
APACHE II score	9.85 ± 5.97	9.51 ± 6.02	10.92 ± 5.72	0.080
EuroScore	5.64 ± 2.74	5.57 ± 2.81	5.88 ± 2.49	0.399
Post-operative blood transfusion, *n* (%)	110 (36.54)	74 (32.46)	36 (49.32)	0.012
**Post-operative laboratory tests**				
Hemobilirubin, μmol/L	38.58 ± 22.28	39.13 ± 21.97	36.86 ± 23.32	0.450
Blood albumin, g/L	33.96 ± 5.24	34.09 ± 5.43	33.56 ± 4.61	0.457
BUN, mmol/L	12.15 ± 4.70	11.73 ± 4.48	13.46 ± 5.15	0.006
CRE, μmol/L	158.96 ± 102.93	149.34 ± 95.66	189.00 ± 118.69	0.004
eGFR, ml/min/1.73 m^2^	54.71 ± 27.67	58.09 ± 28.39	44.15 ± 22.36	<0.001
Normal group (eGFR >90)	45 (14.95%)	43 (95.56%)	2 (4.44%)	
Mild group (eGFR 60–90)	73 (24.25%)	57 (78.08%)	16 (21.92%)	
Moderate group (eGFR 30–60)	124 (41.20%)	89 (71.77%)	35 (28.23%)	
Severe group (eGFR <30)	59 (19.60%)	39 (66.10%)	20 (33.90%)	0.003
cTNT, ng/ml	0.56 (0.38, 1.06)	0.53 (0.36, 0.94)	0.71 (0.44, 1.45)	0.071
NT-proBNP, log transformed pg/ml	6.55 ± 1.07	6.48 ± 1.01	6.77 ± 1.20	0.040
Hemoglobin, g/L	9.70 ± 4.06	9.83 ± 4.57	9.29 ± 1.60	0.321
Blood sodium, mmol/L	139.23 ± 3.99	139.17 ± 4.37	139.88 ± 4.05	0.054
Blood potassium, mmol/L	3.81 ± 0.60	3.86 ± 0.62	3.63 ± 0.46	0.714
Blood calcium, mmol/L	0.94 ± 0.19	0.95 ± 0.22	0.91 ± 0.08	0.022
**Post-operative sedatives**				
Midazolam, *n* (%)	84 (27.91)	50 (21.93)	34 (46.58)	<0.001
Dexmedetomidine, *n* (%)	261 (86.71)	192 (84.21)	69 (94.52)	0.024
Remifentanil, *n* (%)	116 (38.54)	82 (35.96)	34 (46.58)	0.105
Fentanyl, *n* (%)	5 (1.66)	5 (2.19)	0 (0.00)	0.453
Morphine, *n* (%)	137 (45.51)	93 (40.79)	44 (60.27)	0.004
Propofol, *n* (%)	269 (89.37)	200 (87.72)	69 (94.52)	0.155

*Data presented as mean ± SD for continuous variables and percentage for discontinuous variables. CABG, coronary artery bypass grafting; CPB, cardiopulmonary bypass; APACHE, acute physiology and chronic health evaluation; BUN, blood urea nitrogen; CRE, serum creatinine; eGFR, estimated glomerular filtration rate; NT-proBNP, N-terminal prohormone of brain natriuretic peptide*.

### Predictors of POD

**Figure 2:**
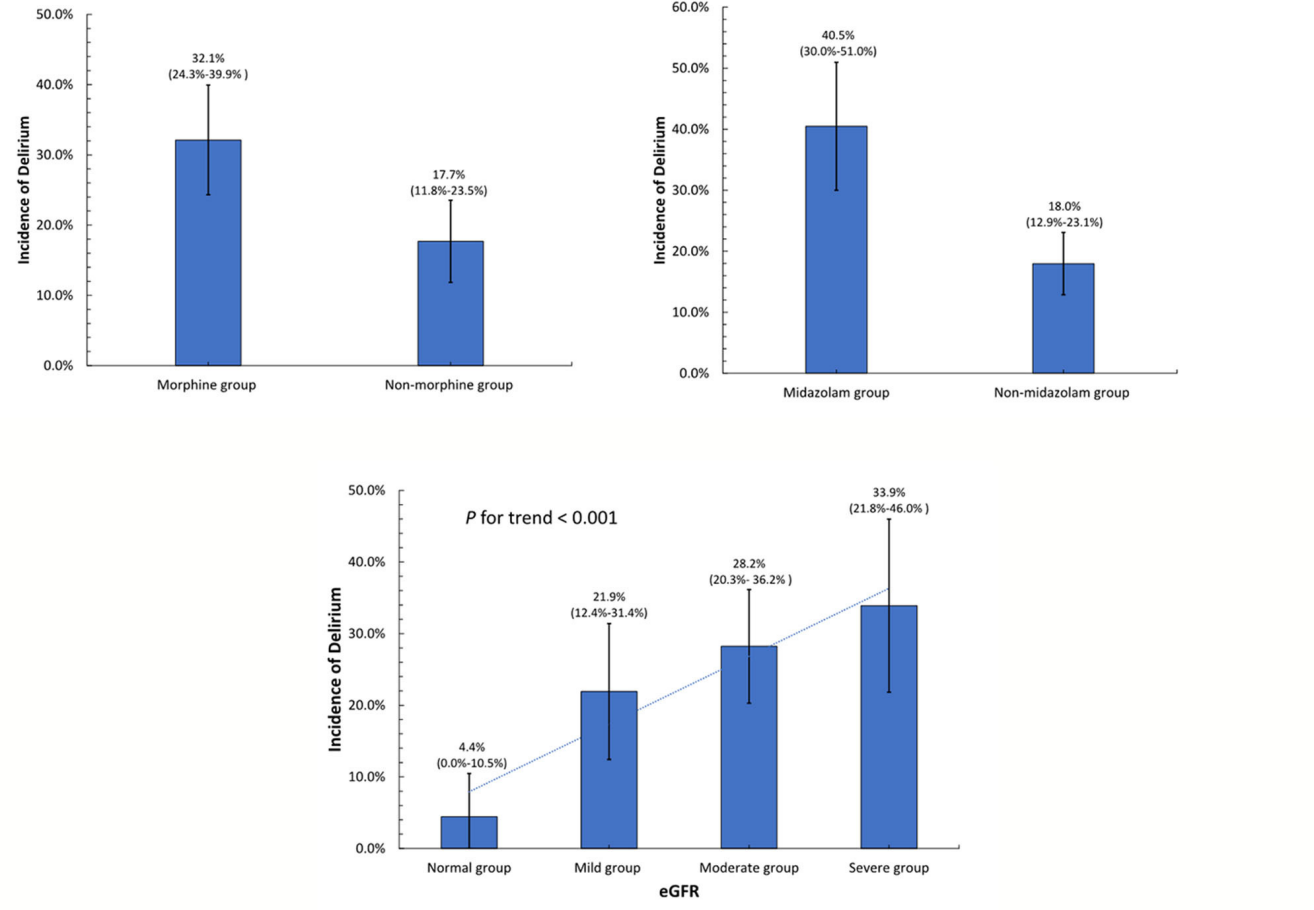
Incidence of post-operative delirium in different groups divided by the independent determinants.

The results of the univariate and multivariate logistic regression analysis for the predictors of POD are presented in Table 2. In the univariate analysis, variables with a *p-*value < 0.1 were included in the multivariable analysis. In the multivariate analysis, a lower eGFR (OR 0.84, 95% CI 0.74–0.94, *p* = 0.003), post-operative use of midazolam (OR 2.37, 95% CI 1.33–4.23, *p* = 0.004), and post-operative use of morphine (OR 1.87, 95% CI 1.07–3.29, *p* = 0.029) were independent predictors of the development of POD among AAD patients. Figure 2 shows the incidence of POD in different groups divided by the independent predictors. We found that patients who used morphine after surgery presented a higher incidence of POD than those who did not use morphine (32.12 vs. 17.68%, *p* = 0.004). In addition, patients who received midazolam had a higher incidence of POD than those who did not (40.48 vs. 17.97%, *p* < 0.001). Patients were classified into four groups according to eGFR to assess renal function after surgery. The incidence of POD showed a significant increase as renal function declined (*p* for trend is <0.001).

**Table 2:** Perioperative risk factors for POD identified by the logistic regression analysis.

**Variables**	**Univariable analysis**			Multivariable analysis^*^		
	**OR**	**95% CI**	***P*-value**	**OR**	**95% CI**	***P*-value**
Duration of surgery, min	1.00	1.00–1.01	0.089	–	–	–
APACHE II score	1.04	1.00–1.09	0.082	–	–	–
Post-operative blood transfusion, *n* (%)	2.03	1.19–3.46	0.010	–	–	–
BUN, mmol/L	1.08	1.02–1.14	0.009	–	–	–
NT-proBNP, log transformed pg/ml	1.29	1.01–1.65	0.042	–	–	–
Blood sodium, mmol/L	1.07	1.00–1.14	0.056	–	–	–
Blood calcium, mmol/L	0.01	0.00–0.33	0.011	0.04	0.00–1.83	0.100
eGFR, per 10 ml/min/1.73 m^2^ increase	0.81	0.73–0.91	<0.001	0.84	0.74–0.95	0.005
Midazolam	3.10	1.78–5.42	<0.001	2.35	1.31–4.22	0.004
Morphine	2.20	1.29–3.77	0.004	1.83	1.04–3.24	0.037

*OR, odds ratio; APACHE, acute physiology and chronic health evaluation; BUN, blood urea nitrogen; NT-proBNP, N-terminal prohormone of brain natriuretic peptide; eGFR, estimated glomerular filtration rate*.

* *The model was adjusted by age and gender*.

### Early Outcomes

The comparison of early outcomes during hospitalization between the delirium group and the non-delirium group is shown in Table 3. Patients with POD had a longer ICU stay (11.52 vs. 7.22 days, *p* < 0.001) and hospital stay (23.99 vs. 18.91, *p* = 0.007) than those without POD. In addition, patients with POD had higher medical expenses than those without POD (48.82 vs. 37.66 thousand dollars, *p* < 0.001). The in-hospital mortality rate was higher in the delirium group than in the non-delirium group, but the difference was not statistically significant (6.85 vs. 4.82%, *p* = 0.502).

**Table 3:** The early outcomes of patients with and without POD.

	**Delirium group (*n* = 73)**	**Non-delirium group (*n* = 228)**	**Mean difference**	**95% CI**	***P*-value**
Post-operative ICU LOS, days	11.52 ± 10.14	7.22 ± 8.17	4.30 ± 1.19	1.97–6.64	<0.001
Post-operative hospital LOS, days	23.99 ± 16.18	18.91 ± 13.00	5.08 ± 1.86	1.41–8.47	0.007
Hospital costs, thousand dollars	48.82 ± 23.95	37.66 ± 17.12	11.16 ± 2.55	6.14–16.19	<0.001
In-hospital mortality, *n* (%)	5 (6.85)	11 (4.82)	2.03%	0.044–0.085	0.502

*Data presented as mean ± SD for continuous variables and percentage for discontinuous variables. LOS, length of stay; ICU, intensive care unit*.

## Discussion

In this study, we found that 24.25% of patients with AAD developed POD during the ICU stay. A lower eGFR and the use of midazolam and morphine after surgery were independent predictors of POD. As a result, patients with POD had longer ICU stays and hospital stays and higher medical expenses than those without POD.

Because AAD is a very emergent and life-threatening disease, most surgeons pay attention to mortality while ignoring post-operative neurological symptoms. However, with improvements in surgical techniques, an increasing number of patients survive after surgery. Thus, post-operative morbidity should be emphasized, as it may directly influence patient outcomes and quality of life. As one of the most severe and complex surgical complications of cardiac vascular disease, POD was frequently observed in our clinical practice. However, although several studies have reported the incidence and risk factors for POD after cardiac surgeries, few have focused on AAD patients. In previous studies, the reported incidence of POD after cardiac surgery varied between 3 and 17.3% (1–4, 20). Gaul et al. found that POD is the most common neurological complication in patients with AAD, accounting for 31.9% of all neurological complication events (10). Liu et al. (14) also reported that the incidence of POD in 100 AAD patients reached 34%. Shi et al. (15) stated an incidence of 45.95% in 148 AAD patients. These studies indicated a higher incidence of POD among AAD patients than among other patients. However, these investigations were limited by small sample sizes. Our study enrolled more than 300 AAD patients and collected detailed data to show that POD affected up to 24.25% of AAD patients after surgery and could help raise awareness of POD management for AAD patients in clinical practice.

Because the mechanism of delirium is unknown, an effective treatment is still lacking at present. Many studies have attempted to identify modifiable and non-modifiable risk factors to detect and prevent the development of delirium early. Clinical practice guidelines describe that benzodiazepine use and blood transfusions are the only two modifiable risk factors for delirium in critically ill adults with strong evidence support (21). In our study, we only found that post-operative blood transfusion was associated with POD in the univariate analysis, but it was not significant in the multivariate analysis. However, avoiding blood transfusions and receiving lower levels of hemoglobin is still controversial (22). In addition, we also found that the use of midazolam and morphine were independent risk factors for POD in AAD patients. Our results also confirmed the associations between delirium and benzodiazepine and opioid use in AAD patients. Furthermore, we identified that a lower eGFR was an independent risk factor for POD in AAD patients. A systematic review including 34 articles demonstrated that the association between renal dysfunction and delirium after on-pump cardiac surgery was inconclusive (23). Our study showed a positive relationship between post-operative renal dysfunction and the development of POD among the AAD population. The incidence of POD significantly increased as the degree of renal function declined. In contrast to previous studies involving patients with other diseases, we did not find any relationship between older age and delirium in AAD patients (23). Furthermore, two studies that included AAD patients showed that age is not an independent risk factor for POD, which was consistent with our results (14, 24).

Many studies have documented that POD is associated with several negative clinical consequences, including major post-operative complications, longer hospital stays with increased costs, and higher mortality (12, 13, 25). However, despite the emergent nature of AAD and high mortality in patients with AAD, only a few studies have focused on POD in these patients. In our study, we further compared the early outcomes of patients with and without POD. The results showed that patients with POD had a longer ICU length of stay and longer hospital length of stay than those without POD. As a consequence, the hospital costs for patients with POD are much higher than those for patients without POD. Contrary to previous studies on other populations, our study showed that the mortality rate was higher in the delirium group than in the non-delirium group, but the difference was not statistically significant. This result might be influenced by the exclusion of patients who were deeply sedated or in a coma, who were the most severe patients, which may underestimate the reported mortality rate. Although we did not find a positive association between POD and post-operative hospital mortality in this study, the longer hospital stay and higher medical costs showed that POD burdens patients and hospitals to some extent. Further prospective studies and follow-up evaluations are required to assess mid-term and long-term patient outcomes.

We acknowledge the potential limitations of our study. First, this study was limited by its observational and retrospective nature. Second, our participants were from a single hospital, so the generalizability of the findings remains to be confirmed. Third, the incidence of delirium might be underestimated as a result of missed diagnoses because delirium status changes over time. However, we also used olanzapine as a proxy for the diagnosis of delirium. Further studies are required to conduct a prospective and multicenter investigation to elucidate the prevalence and clinical outcomes of POD among AAD patients.

## Conclusion

In summary, our findings showed that patients with AAD had a higher incidence of POD during the ICU stay than those without AAD. Patients with lower eGFRs who used midazolam and morphine after surgery were more susceptible to developing POD. Patients who developed POD had a longer ICU stay and hospital stay with higher hospitalization costs than those without POD. More attention should be paid to POD in patients with AAD. Early screening, such as for renal dysfunction, and prevention by using nurse-led sedation scales and protocols to minimize the use of sedative medication might contribute to improving the clinical outcomes of these patients during and after the ICU stay.

## Data Availability Statement

The raw data supporting the conclusions of this article will be made available by the authors, without undue reservation.

## Ethics Statement

The studies involving human participants were reviewed and approved by Zhongshan hospital, Fudan University. Written informed consent for participation was not required for this study in accordance with the national legislation and the institutional requirements.

## Author Contributions

YZ and SC initiated the study. YZ, SC, XZ, WP, ZL, and CW contributed to the design of the study. WP, JZheng, and JZhong contributed to the data collection. JG, ML, and SC contributed to the data analysis and interpretation. YZ, SC, and JL drafted the first manuscript. All authors contributed to manuscript revisions, read and approved the final version of the manuscript. All authors agree to be accountable for the content of the work.

## Conflict of Interest

The authors declare that the research was conducted in the absence of any commercial or financial relationships that could be construed as a potential conflict of interest.
